# Assessment of tissue-specific accumulation, elimination and toxic effects of dichlorodiphenyltrichloroethanes (DDTs) in carp through aquatic food web

**DOI:** 10.1038/s41598-017-02612-4

**Published:** 2017-05-23

**Authors:** Shanshan Di, Ruiquan Liu, Zhongnan Tian, Cheng Cheng, Li Chen, Wenjun Zhang, Zhiqiang Zhou, Jinling Diao

**Affiliations:** 1 Beijing Advanced Innovation Center for Food Nutrition and Human Health, Department of Applied Chemistry, China Agricultural University, Yuanmingyuan west road 2, Beijing, 100193 China; 2 Department of Applied Chemistry, China Agricultural University, Yuanmingyuan West Road 2, Beijing, 100193 China

## Abstract

Microcosms containing DDT spiked-sediment, *Tubifex tubifex* and carp (*Cyprinus carpio*) were constructed to simulate a freshwater system. The accumulation, elimination and toxic effects of DDT (p,p’-DDT, o,p’-DDT), and its metabolites DDD (p,p’-DDD, o,p’-DDD) and DDE (p,p’-DDE, o,p’-DDE) were studied in *T. tubifex* and carp. Tissue/organ distributions of DDTs were also investigated in carp. The bioaccumulation and elimination of DDT differed in *T. tubifex*, carp and its tissues/organs. Unimodal or bimodal distributions were observed, and the concentrations of DDT metabolites (DDD and p,p’-DDE) increased over time. The carp organ with the highest concentrations of DDTs (DDT, DDD and DDE) was the gill. The largest mass distribution of DDTs was also in gill, followed by muscle and gastrointestinal tract. Maximum levels of DDTs in whole carp and carp muscle were 161 and 87 ng/g, respectively; therefore, the levels of DDTs observed in carp in this study were insufficient to constitute a health concern if present in fish for human consumption. Significant changes were observed in some biomarkers, including superoxide dismutase, catalase, glutathione-S-transferase, glutathione, and carboxylesterase, in *T. tubifex* and carp tissues during DDT exposure. Tissue-specific accumulation of DDTs in carp can be a key indicator of exposure to environmentally relevant concentrations.

## Introduction

Dichlorodiphenyltrichloroethane (DDT) is a toxic, ubiquitous, and persistent bioaccumulative organochlorine pollutant of the global environment^1, 2^. Technical grade DDT contains 77.1% p,p’-DDT, 14.9% o,p’-DDT, 4% p,p’-DDE, 0.1% o,p’- DDE, 0.3% p, p’-DDD, and 0.1% o,p’-DDD^3^. DDT can be transformed to DDD and DDE under anaerobic and aerobic conditions, respectively, through biotic or abiotic processes^4^. Exposure to DDT and DDE may be associated with adverse health outcomes, including breast cancer, diabetes, and spontaneous abortion^5^. According to the Canadian Sediment Quality Guidelines for the protection of aquatic life, the effects range-low (ERL) values for DDE, DDD, DDT and DDTs in sediments are 2.2, 2, 1, and 1.58 ng/g, respectively^6^. The standard limits for DDTs in centralized surface drinking water is 1000 ng/L according to environmental quality standards for surface water in China^7^.

Sediment is the main source of contaminants in aquatic ecosystem and the concentration of DDTs reported in sediment differs among regions. In Palos Verdes, 28600 ± 2970 ng/g dry weight (68% p,p’-DDE, 13% o,p’-DDE) was reported^8^, while sediments from Xnoha and Mocu water bodies had maximum concentrations of DDTs of 28.9 and 27.6 μg/g dry weight, respectively, in 2011^9^. In Haling Bay water, the p,p’-DDT level was 0.18 ng/L, while that in sediment was 23.9 ng/g dry weight. Pollutions transformation (4.6 kg/year) and diffusion from sediment to water (1.8 kg/year) are key processes in Hailing Bay sediment, accounting for 57% and 22% of p,p’-DDT emitted, respectively^10^.

The accumulation and degradation of DDTs also differ among animal tissues/organs. In the Pink salmon (*Oncorhynchus gorbuscha*) and Chum salmon (*Oncorhynchus keta*) in the southern Sea of Okhotsk in 2012 and 2013, the order of DDE concentrations in fish organs were eggs < muscles < hepatopancreas < male gonads^11^. Silva *et al*.^12^ reported that DDTs concentrations were 6.90–2405.94 ng/g fat and 2.47–174.29 ng/g fat in the muscle tissue of croakers and mullets in Guanabara Bay (2011), with higher concentrations of o,p’-DDD compared with other forms. Adipose tissue can sequester DDTs and protect other tissues/organs from DDTs overload^13^. Most importantly, the bioaccumulation patterns of DDTs in fish tissues may be useful as effective indicators of environmental DDTs contents, and tissue-specific accumulation of DDTs can be used as a key indicator of chronic exposure. However, the bioaccumulation and tissue distribution of DDTs in carp (*Cyprinus carpio*) from aquatic food web is indistinct.

Evaluation of the biological effects of contaminants is essential for ecological risk assessment, and sediment tests are recommended for this purpose. Biomarker responses in lower complexity organisms may serve as early warning signals of the effects of bioavailable toxic chemicals before manifestation of more severe pathological conditions^14^. DDTs can affect the redox status and reproduction of cells, and cause genotoxic effects^15–18^. Exposure of *Dictyostelium discoideum* amoebae to 732.5 μg/L DDX (p,p’-DDT, p,p’-DDD and p,p’-DDE) in water solution, reactive oxygen species (ROS) increased and the glutathione (GSH) content significantly decreased^14^; however, no harmful or sublethal effects were observed in amoebae in sediments containing the same level of DDX (732.5 μg/kg)^14^.

The objective of this study was to describe the accumulation, distribution, elimination, and biological effects of DDTs in carp tissues/organs in the context of an aquatic food web. The results have the potential to improve our understanding of food web responses to DDT exposure.

## Results and Discussion

### Bioaccumulation of DDTs in *Tubifex tubifex* and carp (*Cyprinus carpio*)

**Figure 1:**
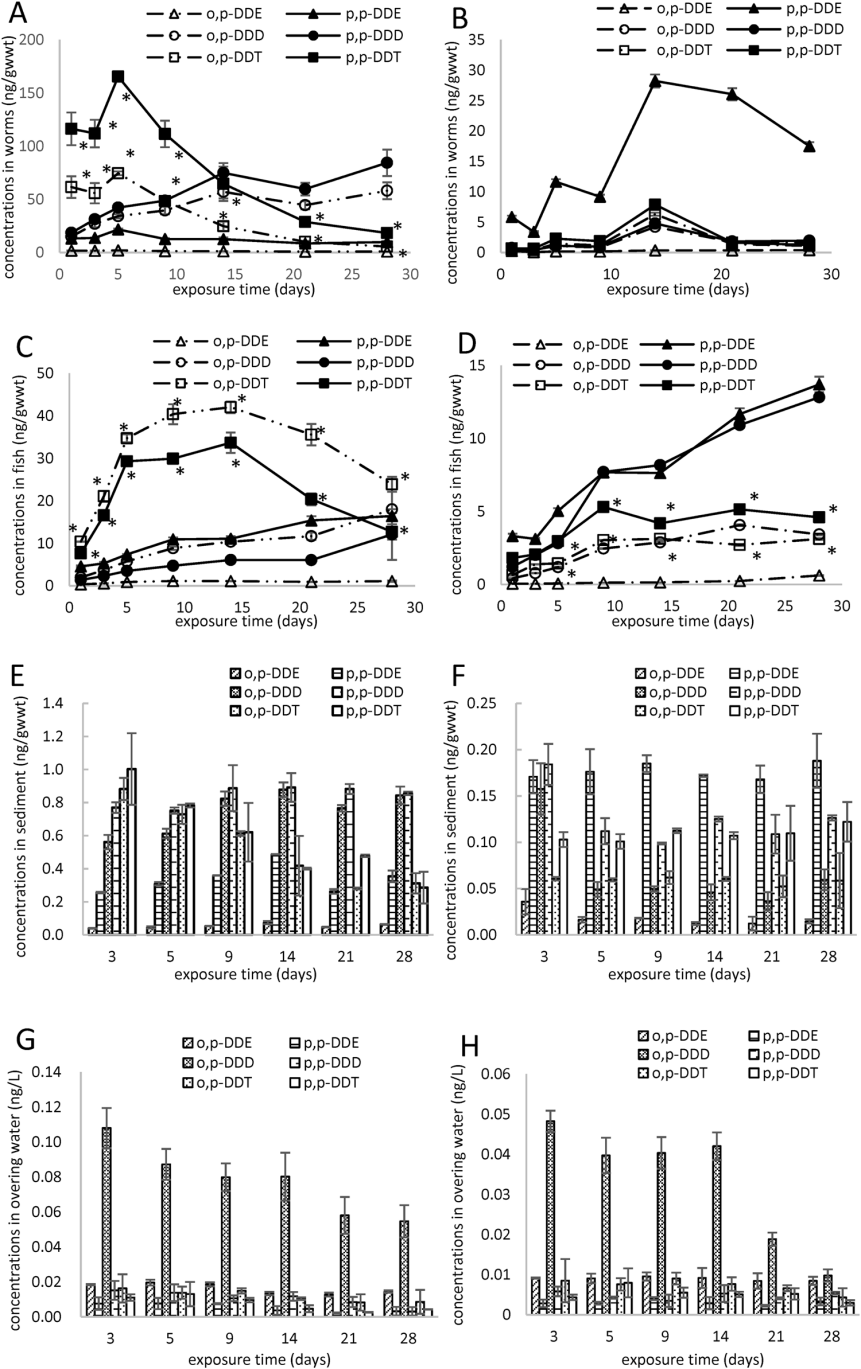
The bioaccumulation of DDTs in *T. tubifex* (**A**,a), carp (**B**,b), sediment (**C**,c) and overlying water (**D**,d) in the treatment group and control group. *Indicates significant difference between p,p’-DDT and o,p’-DDT (*p* < 0.05, S-N-K test).

The results of analysis of bioaccumulation of DDTs in *T. tubifex* are shown in Fig. 1A. It showed rapid accumulation of p,p’-DDT and o,p’-DDT until the fifth day, and reached maximum levels of 165.5 ± 2.7 and 74.6 ± 1.8 ng/g_wwt_, respectively. Subsequently, the concentrations of DDT in *T. tubifex* decreased, and only 18.4 ± 2.2 and 5.6 ± 0.1 ng/g_wwt_ of p,p’-DDT and o,p’-DDT, respectively, were detected on day 28. The concentrations of p,p’-DDT were significantly higher than those of o,p’-DDT. The changes in DDE concentrations in *T. tubifex* were similar to those of DDT, with maximum values of 21.7 ± 0.8 ng/g_wwt_ for p,p’-DDE and 2.1 ± 0.2 ng/g_wwt_ for o,p’-DDE on the fifth day. The high DDTs concentrations accumulated in *T. tubifex* may have significant environmental implications given that they are prey for animals at higher trophic levels. In the control group, the DDTs concentrations in *T. tubifex* (Fig. 1B) were significantly lower than those in the treatment group except for p,p’-DDE. The concentrations of p,p’-DDE were the highest among DDTs in *T. tubifex*, reaching a maximum level of 28.2 ± 1.1 ng/g_wwt_ on the 14th day, and then decreasing gradually. The changes in DDT and DDD concentrations were similar to that of p,p’-DDE. The differences were caused by varying DDT concentrations in the sediment.

The changes in concentrations of DDTs in carp are shown in Fig. 1C. Carp could be exposed to DDTs through direct contact with spiked sediment, occasional ingestion of suspended particulate matter, eating worms, and breathing through the gills. The DDT concentrations in carp increased rapidly and reached a steady state on the 9th day, with 57.3 ± 3.1 and 39.1 ± 0.3 ng/g_wwt_ for p,p’-DDT and o,p’-DDT, respectively. After 21 days, the DDT concentrations decreased. The concentrations of p,p’-DDT in carp were significantly higher than those of o,p’-DDT, consistent with our finding on *T. tubifex*. The concentrations of DDD and DDE increased over time and their constituents were present in levels of descending concentration as follows: o,p’-DDD > p,p’-DDD ≥ p,p’-DDE > o,p’-DDE in carp, and p,p’-DDD > o,p’-DDD > p,p’-DDE > o,p’-DDE in *T. tubifex*. Biomagnification was evaluated by the concentration of the test substances in carp relative to those in their prey, *T. tubifex*, with biomagnification factor (BMF) values > 1 indicating the occurrence of biomagnification^8^. The BMFs indicated that the contaminated *T. tubifex* may pose a risk for its predators (Fig. S1A). Wang *et al*. reported that DDT was biomagnified in the marine food chain from copepods (*Acartia erythraea*) to fish (mangrove snappers *Lutjanus argentimaculatus*)^19^. Also, hexachlorobenzene (HCB) was biomagnified in sticklebacks by accumulating HCB from the contaminated *T. tubifex* worms^20^. Exposed carp may also represent a contamination source for their predators. Leung *et al*. reported that DDTs concentrations were elevated in carnivorous freshwater (1742 lg/kg, lipid weight (l.w.)) relative to omnivores (42.5 lg/kg, l.w.)^21^. In the control group, the DDTs concentrations in carp (Fig. 1D) were significantly lower than those in the treatment group, with p,p’-DDD and p,p’-DDE higher than other DDTs and increasing over time. The concentrations of DDT increased until the 9th day, and then remained stable.

In the treatment group, the DDT concentrations in sediment decreased gradually (Fig. 1E). The concentrations of DDD increased rapidly and remained at a high level (about 0.83 ng/g_wwt_), which were higher than those in control sediment (Fig. 1F). The changes of DDE concentrations in sediment showed an “N” trend. These changes may be caused by the combination effect of *T. tubifex*, carp and microorganisms. It has been proven that, the existence of *T. tubifex* can accelerate the dissipation of isocarbophos and hexachlorocyclohexanes (HCHs) in sediments^22, 23^. The higher DDD concentrations in sediment may be due to the biotransformation of carp, which conceivably produces feces containing more DDD. Furthermore, the bowel movement of carp might be promoted after exposure to DDTs-contaminating *T. tubifex*, and produced more feces. It is worth noting that DDTs in feces could be utilized by fish or some benthic organisms, leading to further exposure. Moreover, the increased DDD and DDE concentrations may pose an additional risk. However, no significant changes of DDD or DDE concentrations were identified in the control sediment (Fig. 1F). The concentrations of DDT decreased significantly on the third day, and then changed little until the end of the experiment. The physicochemical properties of sediment may affect the bioavailability of substances. Reports suggest that increased sand and organic content in sediments may provide additional adsorption sites for some substances, leading to increased retention in sediments and reduced bioavailability^22, 24^.

The changes of DDTs concentrations in overlying water were similar in the treatment (Fig. 1G) and control (Fig. 1H) groups, and decreased over time, however, the DDTs concentrations in the treatment group (Fig. 1G) were significantly higher than those in the control group (Fig. 1H), due to the different DDT concentrations in sediment.

### Bioaccumulation of DDTs in carp tissues/organs

Carp can accumulate and concentrate DDTs in their tissues to levels several orders of magnitude above those in the polluted environment. The deposition or localization, and in some cases metabolism, of DDTs depend on the characteristics of particular tissues and organs, and may influence toxicity^25^. Tissue-specific accumulation of DDTs can be a key indicator of chronic exposure. DDT and its metabolites (DDE and DDD) are lipid-soluble compounds. Once absorbed, they are readily distributed to all body tissues via the lymph and blood^26^. Their uptake into tissues is a function of blood flow, tissue-specific lipid content, and the partition coefficient of DDTs between blood and lipids in specific tissues/organs^26^.

**Figure 2:**
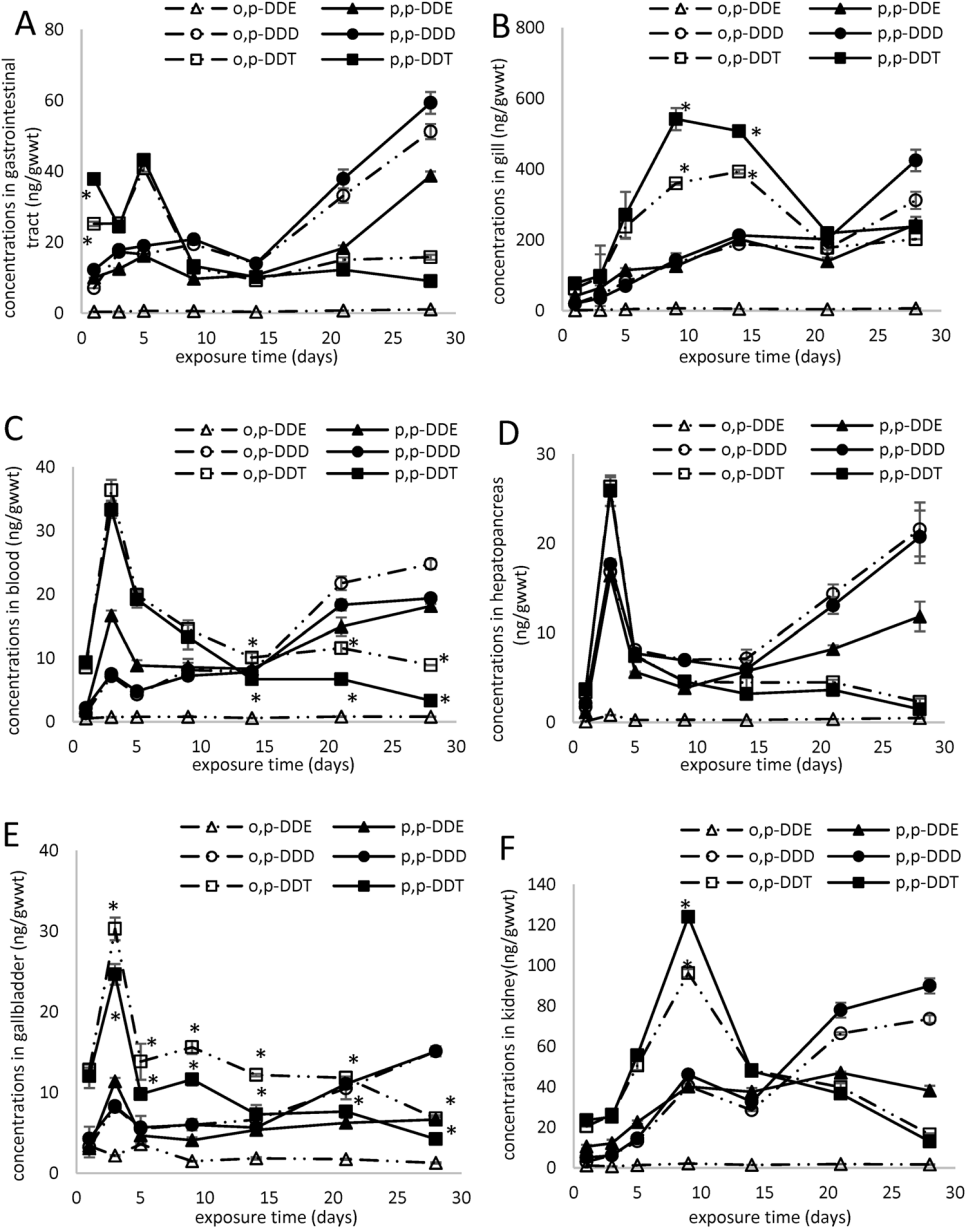
The accumulation of DDTs in gastrointestinal tract (**A**), gill (**B**), blood (**C**), hepatopancreas (**D**), gallbladder (**E**) and kidney (**F**) in carp in the treatment group. *Indicates significant difference between p,p’-DDT and o,p’-DDT (*p* < 0.05, S-N-K test).

Gastrointestinal absorption via the intestinal lymphatic system has a major role in the uptake of DDT in organisms^26^. In food web, dietary intake seems to be a significant route of carp uptake of DDTs through the gastrointestinal tract, and the accumulation profiles for DDT in this organ indicated that the process proceeded in two steps (Fig. 2A). During the first 5 days, DDT concentrations increased rapidly, reaching maximum levels of 43.2 ± 0.5 and 40.9 ± 0.9 ng/g_wwt_ for p,p’-DDT and o,p’-DDT, respectively; subsequently, DDT concentrations decreased slowly. The profiles of DDD and DDE concentrations showed an “N” trend. A decrease occurred on the 14th day, then the concentrations of DDD and DDE increased sharply, with p,p’-DDD ≥ o,p’-DDD > p,p’-DDE > o,p’-DDE. In the control group, the DDTs concentrations in gastrointestinal tract (Fig. S2a) were significantly lower than those in treatment group. The DDTs concentrations increased over time, and the concentrations of p,p’-DDD and p,p’-DDE were higher than those of others.

The gill is in direct contact with the aquatic environment, and its external position, highly branched structure, and vascular nature highly increased surface area through which large volumes of water pass through the gill surface, making it a target organ for waterborne toxicants^27, 28^. Bioturbation by *T. tubifex* and carp can agitate sediment particulate matter, and increase the levels of DDTs in overlying water. Our results demonstrate that the DDTs concentrations in gills (Fig. 2B) were significantly higher than those in other tissues/organs. Furthermore, the amount of mucus on the gill surface might increase during DDTs exposure, which might improve DDTs contents in gill. It is also possible that dietary DDTs enter the branchial epithelium from the blood, allowing DDTs taken up through the gastrointestinal tract to increase DDTs contents in gill via the body circulation. The concentration of p,p’-DDT and o,p’-DDT reached maximum level on the 9th and 14th day, respectively, and the p,p’-DDT concentrations were significantly higher than those of o,p’-DDT. The concentrations of DDD and DDE increased over time in gill, and reached a maximum at the end of the experiments with p,p’-DDD ≥ o,p’-DDD ≥ p,p’-DDE > o,p’-DDE. In the control group, the DDTs concentrations in gill (Fig. S2b) were significantly lower than those in the treatment group. The DDTs concentrations increased over time, and the concentrations of p,p’-DDE were significantly higher than those of others.

As most substances are distributed via the bloodstream, consequently blood components are exposed, at least initially, to significant concentrations of toxic substances from the environment^25^. Sieber^29^ reported that DDT and some structurally-related compounds, were preferentially absorbed via the intestinal lymphatic system in rats, with some absorption into blood. In blood (Fig. 2C), the concentrations of DDT, DDD, and p,p’-DDE increased sharply and reached a high level on the third day. Subsequently, DDT concentrations decreased over time, while the concentrations of DDD and p,p’-DDE exhibited a “decrease-increase” trend, with significantly higher o,p’-DDT than p,p’-DDT levels. In the control group, the DDTs concentrations in blood (Fig. S2c) were significantly lower than those in the treatment group. The concentrations of p,p’-DDT, DDD and p,p’-DDE increased over time, with concentrations of p,p’-DDD > p,p’-DDE > p,p’-DDT ≥ o,p’-DDD, and those of o,p’-DDT decreased over time in the control group.

Hepatopancreas and kidney are tissues/organs responsible for major metabolic activities including detoxification. DDTs might be transported into these organs from other tissues such as the muscle and gills, for subsequent elimination^28^. Substances taken into the body from gastrointestinal tract are absorbed into the hepatic-portal blood system and pass via the portal vein to the hepatopancreas^25^. The close proximity of blood in the sinusoids to hepatocytes allows efficient exchange of compounds, and consequently substances are taken up very readily into hepatocytes^25^. The membranes of adjacent hepatocytes form the bile canaliculi into which bile is secreted. The bile canaliculi form a network feeding bile into ductules which become bile ducts^25^. In hepatopancreas (Fig. 2D) and gallbladder (Fig. 2E), the DDTs concentrations reached a peak on the 3rd day, and then decreased sharply. After 5 days, the DDT concentrations decreased slowly, while the concentrations of DDD and DDE increased over time. The changes in levels of DDTs observed in hepatopancreas and gallbladder were similar to those identified in blood. The increased concentrations of DDD and DDE might reflect the metabolism and degradation of DDT. Peterson *et al*. reported that, after 6 days’ dietary exposure to DDT (1500 μg/g), DDT, DDD and DDE were detected in the livers of rats, at an approximately 3:5:1 ratio^30^. Meanwhile, DDT can be metabolized by rat liver microsome to a phenolic compound and to a reduced derivative similar to DDD^31^. The concentrations of p,p’-DDT and o,p’-DDT were similar in hepatopancreas (Fig. 2D), while o,p’-DDT concentrations were significantly higher than those of p,p’-DDT in gallbladder (Fig. 2E). No significant difference were observed between p,p’-DDD and o,p’-DDD concentrations. The concentrations of DDD were higher than those of p,p’-DDE and o,p’-DDE. In the control group, the DDTs concentrations in hepatopancreas (Fig. S2d) and gallbladder (Fig. S2e) were significantly lower than those in the treatment group. For hepatopancreas (Fig. S2d), the DDTs concentrations (except o,p’-DDE) reached a peak on the 5th day, and then decreased sharply. After 9 days, the concentrations of DDT, DDD and p,p’-DDE increased over time. The concentrations of p,p’-DDE were the highest, followed by p,p’-DDD. For gallbladder (Fig. S2e), the DDTs concentrations (except o,p’-DDE) reached peak on the 9th day, and the concentrations followed the order: p,p’-DDD > p,p’-DDE > p,p’-DDT > o,p’-DDT > o,p’-DDD. After 14 days, the DDT concentrations decreased slowly, while the concentrations of DDD and p,p’-DDE remained stable.

**Figure 3:**
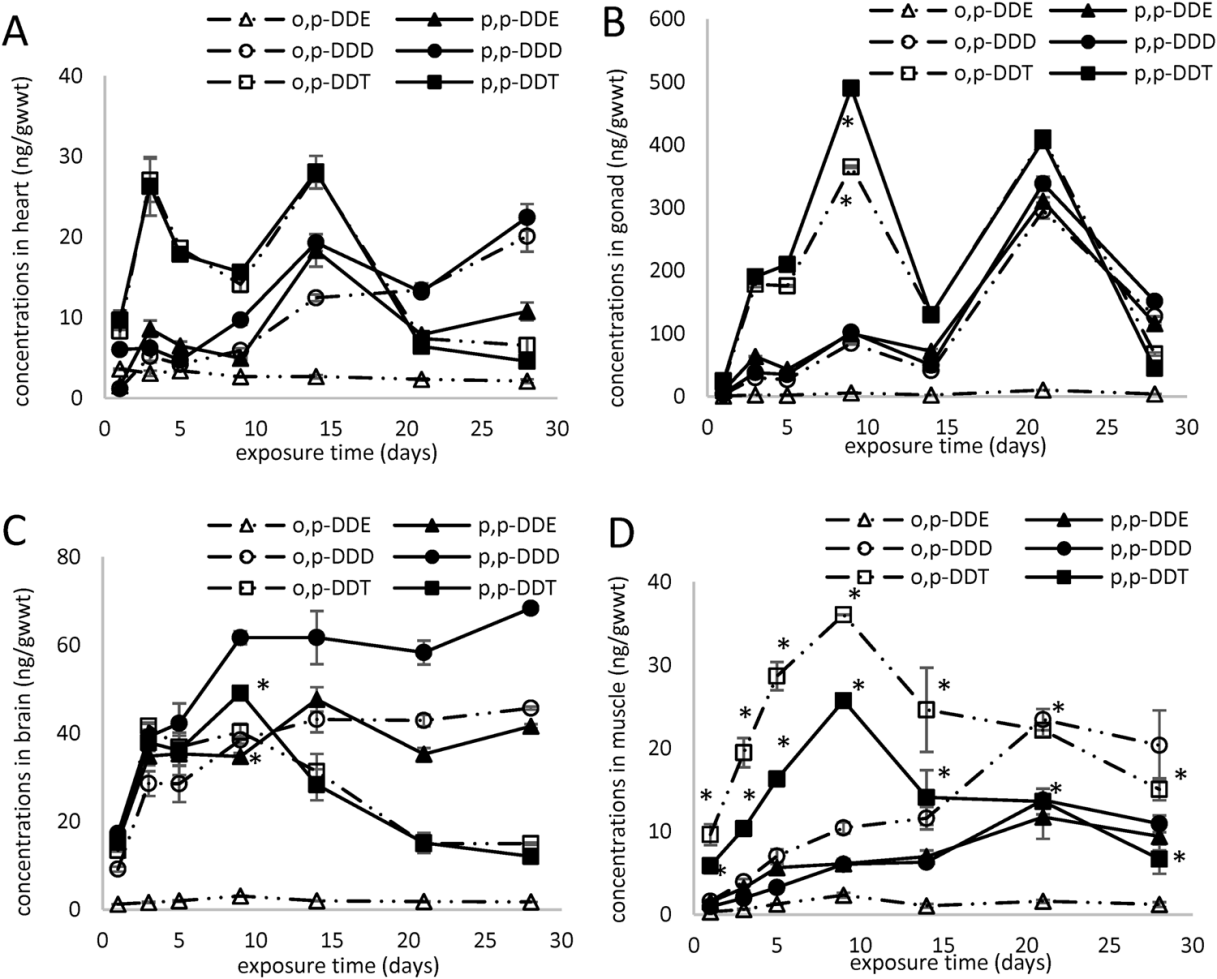
The accumulation of DDTs in heart (**A**), gonad (**B**), brain (**C**) and muscle (**D**) in carp in the treatment group. *Indicates significant difference between p,p’-DDT and o,p’-DDT (*p* < 0.05, S-N-K test).

DDTs might be released from the hepatopancreas cell and gradually redistributed to the kidney. The function of kidney is to filter waste products and toxins out of the blood while conserving essential substances^25^. Anatomically, the kidney is a complex arrangement of vascular endothelial cells and tubular epithelial cells, the blood vessels and tubules being intertwined^25^. The DDTs concentrations in kidney (Fig. 2F) were higher than those in hepatopancreas, which might be explained by the following reasons^25^: (1) the concentration ability of the kidney. After glomerular filtration many substances are reabsorbed from the tubular fluid; (2) active transport of substances by the tubular cells: substances which are actively transported from the blood into the tubular fluid may accumulate in the proximal tubular cells, and the concentrations in tubular cells are higher than those in the blood (Fig. 2C); (3) the lipid content of kidney (2.99 ± 0.42% wwt) was higher than that of hepatopancreas (2.27 ± 0.15% wwt); (4) in some cases hepatic metabolism may be involved followed by transport to the kidney and subsequent toxicity. The DDT concentrations in kidney reached a maximum on the 9th day, when the concentrations of p,p’-DDT (124.0 ± 2.6 ng/g_wwt_) were higher than those of o,p’-DDT (96.2 ± 2.0 ng/g_wwt_); no significant differences were found at other time points. The concentrations of DDD in kidney increased over time until the 9th day; subsequently, following a brief decrease, the concentrations increased rapidly, reaching maxima on the 28th day (89.9 ± 3.7 and 73.5 ± 1.9 ng/g_wwt_ for p,p’-DDD and o,p’-DDD, respectively). The highest concentration of p,p’-DDE (46.9 ± 0.6 ng/g_wwt_) was observed on the 21st day. The changes in o,p’-DDE was similar to those of DDT. In the control group, the DDTs concentrations in kidney (Fig. S2f) were significantly lower than those in the treatment group. The concentrations of p,p’-DDD and p,p’-DDE exhibited an “increase-decrease-increase” trend, while those of DDT, o,p’-DDD and o,p’-DDE increased over time.

Typical bimodal phenomena were observed in heart (Fig. 3A) and gonad (Fig. 3B). In heart, the first and second peak appeared on the 3rd day and 14th days, respectively, while in gonad they were observed on the 9th and 21st days, respectively. We speculated that the appearance of the second peak might be related to absorption via the gastrointestinal tract, and transportation through the bloodstream. Additionally, the concentrations of DDTs in gonad were higher than those in other tissues/organs except gill, which might be due to the physicochemical properties of DDTs, and the lipid content of the gonads (5.03 ± 0.70% wwt). DDT can cause an estrogenic response in rats, leading to thickening of the endometrium and increased in uterus weight^25^. In the control group, the changes in DDTs concentrations in heart (Fig. S3a) were similar to those in gallbladder (Fig. S2e), while the levels of DDTs in gonad (Fig. S3b) increased over time, with p,p’-DDD > p,p’-DDT ≥ p,p’-DDE > o,p’-DDD > o,p’-DDT > o,p’-DDE.

The lipophilicity of DDTs enables them to cross the blood-brain barrier readily without a specific transporter^26^. In carp, DDT accumulation in brain (Fig. 3C) increased until the 9th day reaching 49.1 ± 1.0 ng/g_wwt_ for p,p’-DDT and 40.3 ± 1.8 ng/g_wwt_ for o,p’-DDT, and then decreased with exposure time. The concentrations of p,p’-DDD, o,p’-DDD and p,p’-DDE increased over time, and then changed little after 9 days. At the end of the experiments, the concentrations of p,p’-DDD, o,p’-DDD and p,p’-DDE in brain were 68.4 ± 0.6, 45.7 ± 0.3, and 41.6 ± 0.5 ng/g_wwt_, respectively. The accumulation of o,p’-DDE was similar to that of DDT, with a maximum concentration of 3.1 ± 0.1 ng/g_wwt_ on the 9th day. In the control group, the DDTs concentrations in brain (Fig. S3c) were significantly lower than those in the treatment group, with those of p,p’-DDE the highest.

Substances first enter viscera through feeding, or enter gills through respiration and are then distributed to muscle via blood circulation^32^. In muscle (Fig. 3D), accumulation profiles of DDTs were similar to those of kidney (Fig. 2F), while DDTs concentrations in muscle were lower than those in kidney. Accumulation curves with single peak were observed for DDT and o,p’-DDE, and the concentrations of o,p’-DDT were significantly higher than those of p,p’-DDT. The concentrations of p,p’-DDD, o,p’-DDD and p,p’-DDE increased with exposure time, reaching maxima of 13.8 ± 0.7, 23.5 ± 1.3, and 11.7 ± 2.6 ng/g_wwt_, respectively, on the 21st day. The concentrations of p,p’-DDD were higher than those of o,p’-DDD and p,p’-DDE. In the control group, the DDTs concentrations in muscle (Fig. S3d) were similar to those in whole fish (Fig. 1b).

### Mass distributions of DDTs in carp

The mass distributions of DDTs in carp are presented in Fig. S4A. The mass distributions of p,p’-DDT and o,p’-DDT were higher than those of others, and decreased over time. The mass distributions of p,p’-DDD and o,p’-DDD increased over time, while that of p,p’-DDE increased slowly. The o,p’-DDE mass distribution was negligible. In the control group (Fig. S5a), the changes in the mass distributions of DDTs were similar to those in the treatment group; however, those of p,p’-DDD and p,p’-DDE were higher than those of others.

The mass distribution of total DDTs in the tissues/organs of carp is illustrated in Fig. S4B. The masses of DDTs in heart and gallbladder were negligible. The maximum tissue load was in gill, followed by muscle and gastrointestinal tract. The mass percentages in gill and kidney increased over time, while no significant changes were observed in muscle, gonad or blood. The percentages in brain and hepatopancreas decreased, while those in gastrointestinal tract exhibited a “decrease-increase” trend. These differences among organs may due to features of circulatory systems (e.g., blood and lymph), combined with differing capacities of organs for accumulation, storage and metabolism. In the control group (Fig. S5b), the tissue with the maximum mass distribution was muscle, followed by gastrointestinal tract.

The mass percentages of p,p’-DDT (Fig. S4C) in the tissues/organs of carp followed the order: gill > muscle > gastrointestinal tract > gonad > kidney > brain = hepatopancreas > blood > gallbladder = heart. The percentages of p,p’-DDT in gill increased, while those in muscle, brain, hepatopancreas and blood decreased over time. Minimal changes were observed in kidney and gonad. The percentages in gastrointestinal tract showed a “decrease-increase” trend. The mass percentages of o,p’-DDT (Fig. S4D) in the tissues/organs of carp followed the order: muscle > gill > gastrointestinal tract > gonad > brain > hepatopancreas > kidney > blood > gallbladder > heart. The distribution of o,p’-DDT mass among the tissues/organs was similar to that of p,p’-DDT, except for muscle, which changed little during the exposure time. The order of DDD mass distribution percentages (Fig. S4A and F) was: gill > muscle > gastrointestinal tract > brain > gonad ≥ hepatopancreas > kidney > blood > gallbladder ≥ heart. The changes in DDD were similar to those of o,p’-DDT, except for kidney and blood; the percentages in kidney increased over time, and no significant alterations were found in blood. The distribution percentages of p,p’-DDE (Fig. S4G) and o,p’-DDE (Fig. S4H) mass in different tissues/organs were different, while their changes over time were similar. In the control group (Fig. S5c–h), the tissue with the maximum mass distribution was muscle, followed by gastrointestinal tract. The percentages in muscle decreased over time, while those in gastrointestinal tract increased.

### Elimination of DDTs in *T. tubifex* and carp

**Figure 4:**
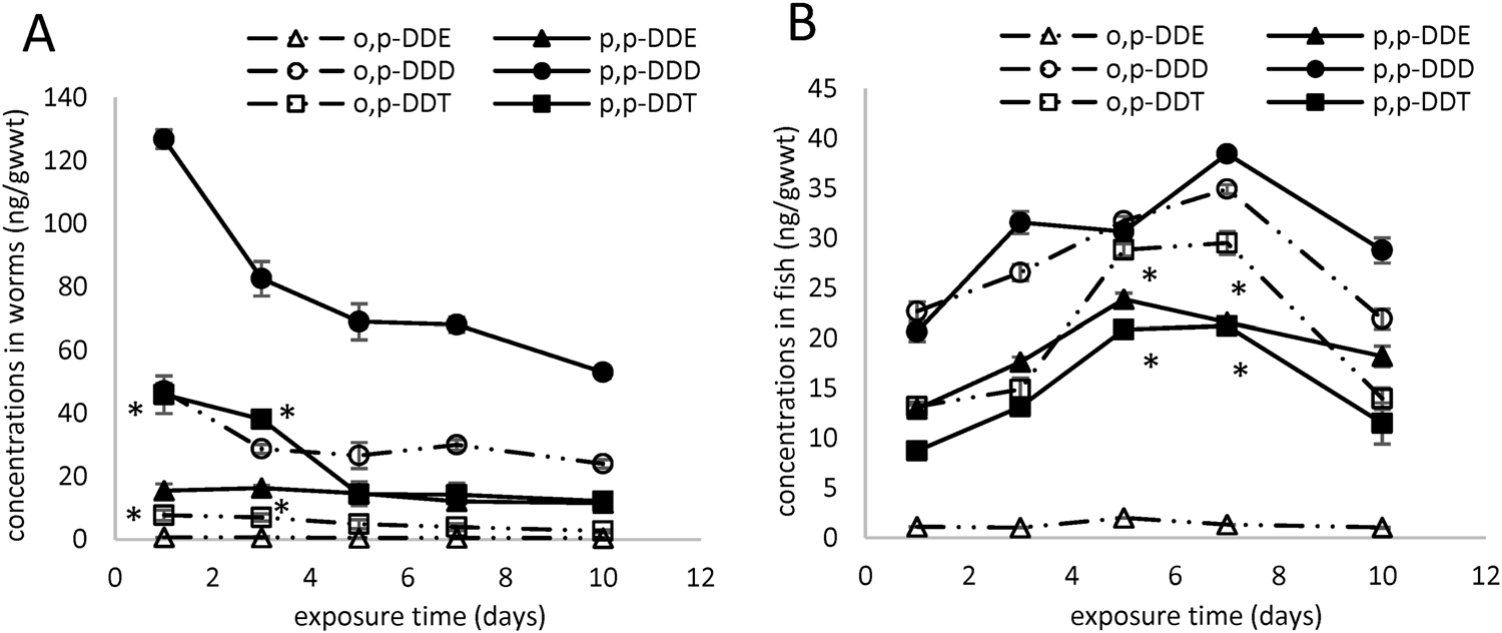
The eilimination of DDTs in *T. tubifex* (**A**) and carp (**B**). *Indicates significant difference between p,p’-DDT and o,p’-DDT (*p* < 0.05, S-N-K test).

The results of the elimination of DDTs in *T. tubifex* and carp are shown in Fig. 4A and B, respectively. The DDTs concentration in *T. tubifex* decreased over time, with elimination efficiencies of 39.5, 25.7, 49.0, 58.2, 65.7 and 73.6% for o,p’-DDE, p,p’-DDE, o,p’-DDD, p,p’-DDD, o,p’-DDT and p,p’-DDT, respectively. The DDTs concentration in carp showed an “increase-decrease” trend, which might be due to the DDTs concentrations in the control sediment. Wang *et al*. reported that there was little depuration of DDT from the fish (mangrove snappers *Lutjanus argentimaculatus*) following its uptake from either aqueous or dietary sources^19^. In addition, the DDT concentrations increased along the food chain (pelagic crustaceans, copepods, euphausiids, amphipods, fish, and seabirds), which indicated that DDT had a lower potential for elimination and was very resistant to metabolic breakdown in fish^19, 33^. The changes in concentrations of DDTs in overlying water were similar in the group with *T. tubifex* and the group with carp, exhibiting little variation, except o,p’-DDD, which decreased over time. The changes in concentrations of DDTs in sediment were similar to those in overlying water, showing little variation, except for o,p’-DDD in the group with *T. tubifex*, which increased over time.

### Elimination of DDTs from carp tissues/organs

DDT could be metabolized by Phase I metabolism (reactions involving oxidation, reduction, and hydrolysis); some metabolites are ultimately excreted in conjugated forms, along with glycine, bile acid conjugates, serine, aspartic acid, or glucuronic acid^26^. The metabolism of DDT can be considered as both a detoxification and an activation reaction^26^. In general, the metabolism of DDTs in animals is similar to that in humans; however, there are also differences between and within species, and between tissues/organs^26^. The ability of carp to eliminate DDTs varied according to tissue/organ (Fig. S6A–j). In the elimination experiments, the DDTs concentrations in gastrointestinal tract (Fig. S6A) and hepatopancreas (Fig. S6D) increased until the 7th day, and then decreased. Similar changes were observed in muscle (Fig. S6J) and gonad (Fig. S6H). Cinier *et al*. reported that, during the depuration period, cadmium bioaccumulation continued in the kidney of carp (*Cyprinus carpio*) after high dose exposure, while no significant changes were observed in liver^34^. These changes might be due to the adsorption and redistribution of DDTs among tissues. The DDT concentrations in other tissues decreased slowly or fluctuated, and the concentrations of DDD and DDE decreased over time. The elimination percentages of DDD and DDE from blood (Fig. S6C) were more than 37.5% and 54.3%, respectively. Those of p,p’-DDD, o,p’-DDD, p,p’-DDE and o,p’-DDE from gallbladder (Fig. S6E) were 64.1%, 66.9%, 50.8% and 24.1%, respectively. At the end of experiments, 63.9% p,p’-DDD, 65.4% o,p’-DDD, 48.7% p,p’-DDE and 47.5% o,p’-DDE were eliminated from kidney (Fig. S6F), and 61.4% p,p’-DDD, 65.5% o,p’-DDD, 52.8% p,p’-DDE and 55.0% o,p’-DDE were eliminated from brain (Fig. S6I). The elimination percentages of o,p’-DDT, p,p’-DDD, o,p’-DDD, and p,p’-DDE in heart (Fig. S6G) were 51.9%, 69.6%, 72.5% and 59.9%, respectively. In the control group (Figs S2 and S3), the changes in DDTs concentrations in the carp tissues/organs were similar to those in the control group in the bioaccumulation experiments.

Elimination routes of DDTs from carp include gill, bile, urine and mucus. The gills are the primary sites of passive diffusion of lipophilic chemicals both into and out of the fish^19^. McKim *et al*. reported that, after rainbow trout were exposed to pentachlorophenol in water, 50% of the dose was eliminated through the gills, 30% in feces and bile, and 20% in urine^35^. Metabolic transformation may also facilitate the elimination of DDT from the body^19^, however, the elimination of DDTs from the gill (Fig. S6B) was slow. The changes in DDTs concentrations in hepatopancreas suggested that the capacity of DDTs to undergo biotransformation in hepatopancreas were greater than their capacity for elimination. The rapid elimination of DDD and DDE from gallbladder and kidney suggested that the hepatic-biliary excretory route and renal pathway might be effective. Moreover, fecal elimination via the digestive system might also contribute.

### Health risk assessment

Potential risks to human health from exposure to DDTs were assessed using two main criteria. The first criterion was the maximum residue limit (MRL)^36^. The MRL for DDTs (containing o,p’-DDT, p,p’-DDT, p,p’-DDD and p,p’-DDE) in aquatic animal products in China is 500 ng/g^37^. In this study, the maximum levels for DDTs (including o,p’-DDT, p,p’-DDT, o,p’-DDD, p,p’-DDD, o,p’-DDE and p,p’-DDE) in whole fish and muscle were 161 and 87 ng/g respectively, which is lower than the MRL for DDTs in China. Also, the maximum concentrations of DDTs in fish were lower than the action levels for DDTs (5.0 mg/kg) developed by the U.S. Food and Drug Administration (U.S. FDA, FDA action levels are indicators of chemical residue levels in fish and shellfish that should not be exceeded for the general population who consume fish and shellfish typically purchased in supermarkets or fish markets that sell products that are harvested from a wide geographic area, including imported fish and shellfish products.)^38^. The second criterion was the acceptable daily intake (ADI) and dietary intake risk (%ADI)^39^. The ADI of DDTs in China is 0.01 mg/kg bw (bw: body weight)^37^. The per capita consumption of aquatic products nationwide was 11.2 kg/year or 0.0307 kg/day (assuming 365 days a year)^40^. The dietary intake risk (%ADI) was calculated from the following equation:

$$ \% {\rm{ADI}}={{\rm{C}}}_{{\rm{m}}}\times 0.0307/({\rm{ADI}}\times 60)\times 100,$$

where C_m_ was the maximum concentration of DDTs in fish or muscle (mg/kg), and 60 was a typical body weight (kg)^39^. %ADI values > 100% indicate unacceptable risk, and a requirement for health monitoring^39^. The calculated dietary intake risks (%ADI) were 0.824% and 0.445% for whole fish and muscle, respectively. Thus, consumption of carp contaminated with the levels of DDTs observed in this study would not be cause for concern for human health.

### Toxic effects assessment

Exposure to DDTs caused significant variation in the concentrations and activities of some enzymes, which directly reflected cell damage in specific tissues/organs. DDTs induced microsomal production of mixed function oxidases that are involved in the catabolism of both xenobiotics and many endogenous hormones^26^. *T. tubifex* can be used as a bio-indicator for the sediment toxicity tests^41^. In carp, the direct exposure of gill to the water makes it the main site of exposure to water contamination and toxicity^42^. Hepatopancreas and kidney are the important organs involved metabolic processes and in the detoxification of xenobiotics. Therefore, some biomarkers were evaluated in *T. tubifex*, and carp gill, hepatopancreas and kidney.

### Superoxide dismutase activity (SOD)

**Figure 5:**
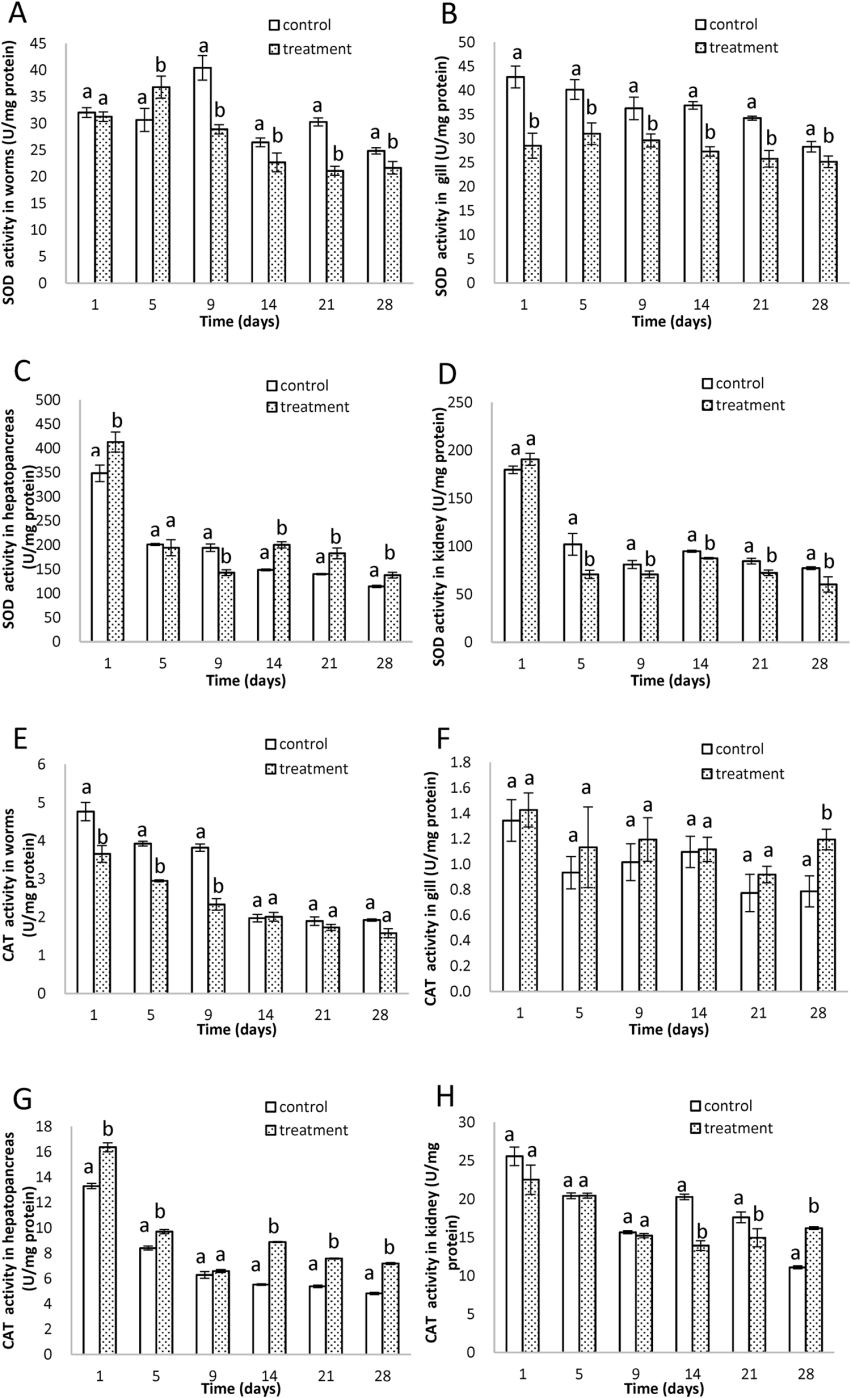
The activities of superoxide dismutase activity (SOD) in *T. tubifex* (**A**), gill (**B**), hepatopancreas (**C**), and kidney (**D**). The activities of catalase activity (CAT) in *T. tubifex* (**E**), gill (**F**), hepatopancreas (**G**), and kidney (**H**). Different letters denote significant difference at the same time point (p < 0.05, S-N-K test), bars are standard deviation (SD). All samples were extracted and analyzed in triplicate.

SOD is the first enzyme to process oxy-radicals, and is sensitive to contaminations stress^43^. In *T. tubifex*, the SOD activity (Fig. 5A) increased significantly on the 5th day, and then decreased significantly until the end of the experiment. Significant decrease was observed in carp gill (Fig. 5B) in the whole exposure time, and similar changes were observed in kidney (Fig. 5D) for the SOD activities. However, the SOD activities were higher significantly in hepatopancreas (Fig. 5C). The induction might be due to the ROS generated in response to increasing concentrations of DDD and DDE. Increased SOD activities can act as a signal of oxidative stress, and the increase probably because the synthesis of new enzymes or the enhancement of pre-existing enzyme levels^43^. The observed decreases indicate that SOD was overwhelmed by ROS, and represent reduced defense capability against oxidative stress^43^.

### Catalase activity (CAT)

The SOD/CAT system provides a first line of defense against oxidative toxicity^44^. The CAT activities decreased significantly during early exposure periods (1, 5 and 9 days) in *T. tubifex* (Fig. 5E), increasing thereafter, and attaining values comparable to those of the control group from 14 days on. In gill (Fig. 5F), significant increase in CAT activity was observed on the 28th day. CAT activities initially increased in hepatopancreas (Fig. 5G), reaching a maximum value on the 14th day and remained elevated until the 28th day. During early exposure periods, no significant changes were found in kidney (Fig. 5H), then levels decreased drastically. The CAT activities were associated with the SOD activities in *T. tubifex* (r = 0.822 (Pearson correlation coefficient r), P = 0.045 (significance test P value)) and hepatopancreases (r = 0.986, P = 0.001). However, the antioxidant defense mechanisms are complex, and other enzyme (e.g. glutathione peroxidase (GPx)) may participate in the catabolism of H_2_O_2_^42^.

### Glutathione-S-transferase (GST) activity and glutathione (GSH) content

**Figure 6:**
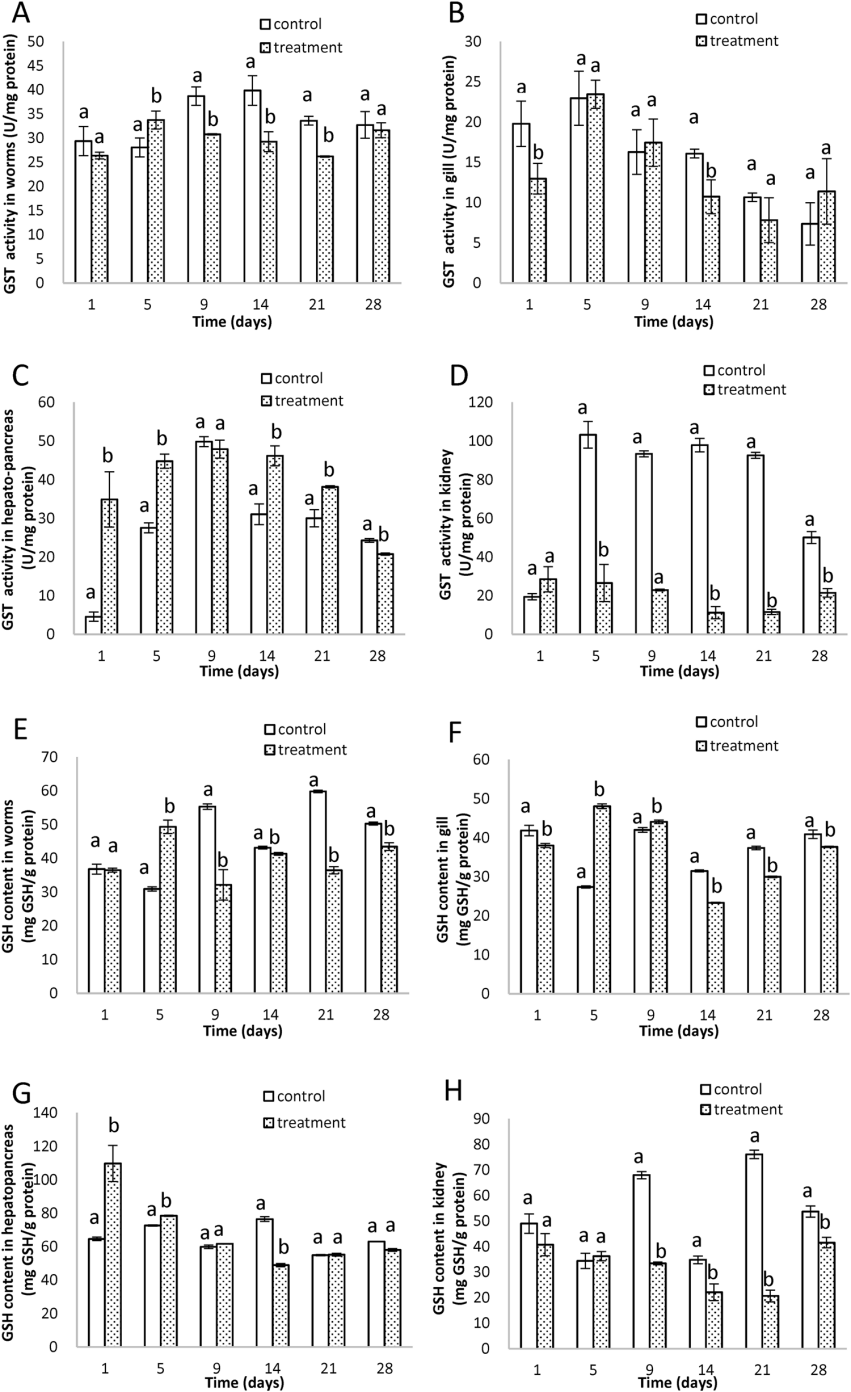
The activities of glutathione-S-transferase (GST) in *T. tubifex* (**A**), gill (**B**), hepatopancreas (**C**), and kidney (**D**). The contents of glutathione (GSH) in *T. tubifex* (**E**), gill (**F**), hepatopancreas (**G**), and kidney (**H**). Different letters denote significant difference at the same time point (p < 0.05, S-N-K test), bars are standard deviation (SD). All samples were extracted and analyzed in triplicate.

GST and GSH participate in antioxidative defenses, and GST is a Phase II detoxification enzyme catalyzing abundant compounds to less toxic substances by conjugating them to GSH^45, 46^. In *T. tubifex*, the GST activities (Fig. 6A) and GSH contents (Fig. 6E) increased significantly on the 5th day, followed by significant decrease. GST activities decreased significantly in carp gill (Fig. 6B) on the 1st and 14th days. Compared to the control group, the GSH contents in gill (Fig. 6F) showed a “decrease-increase-decrease” trend. In hepatopancreas, GST activities (Fig. 6C) increased significantly until the 21st day, and then decreased significantly. For GSH contents (Fig. 6G), significant increase was observed on the 1st day, and significant decrease was observed on the 14th day. The GST activities (Fig. 6D) were significantly inhibited in kidney, and the maximum inhibition was observed on the 14th day. Accordingly, the GSH contents (Fig. 6H) decreased significantly, and the minimum value was observed on the 21st day. The elevation of GST activities and GSH contents reflected the compensatory response and the stimulation of detoxification mechanism. Severe oxidative stress and abundant DDTs suppressed the GST activities and decreased the GSH contents due to oxidative damage, and loss of compensatory and toxic effects^47^. The GST activities correlated significantly with the GSH contents in gill (r = 0.841, P = 0.036) and kidney (r = 0.899, P = 0.015).

### Lactate dehydrogenase activity (LDH)

**Figure 7:**
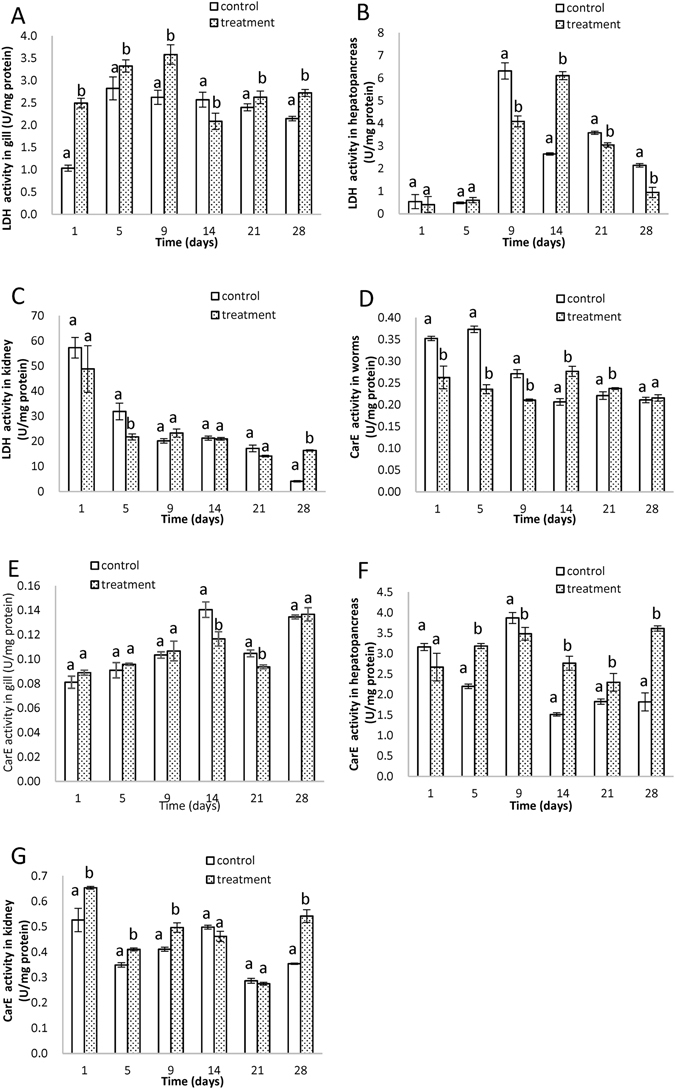
The activities of lactate dehydrogenase (LDH) in gill (**A**), hepatopancreas (**B**), and kidney (**C**). The activities of carboxylesterase (CarE) in *T. tubifex* (**D**), gill (**E**), hepatopancreas (**F**), and kidney (**G**). Different letters denote significant difference at the same time point (p < 0.05, S-N-K test), bars are standard deviation (SD). All samples were extracted and analyzed in triplicate.

LDH, a cytoplasmic biomarker in the glycolytic pathway, is crucial in regulating glycolysis and for normal cellular functions^48^. In *T. tubifex* the LDH activities were too little to measure. In carp gill, the LDH activities increased significantly compared to the control group, except the 14th day (Fig. 7A). Significant decreases were observed in hepatopancreas (Fig. 7B) after 5 days, except the 14th day. In kidney (Fig. 7C), the LDH activities decreased significantly on the 5th day and increased significantly on the 28th day. The elevation and inhibition of LDH activities suggested the DDTs interfered with energy metabolism. The inhibition of LDH activity has been reported in the tissues of *Clarias batrachus* exposed to carbofuran and endosulfan^48, 49^. The inhibition indicated a decline in the catalytic efficiency of LDH, which might be due to the formation of enzyme-inhibitor complex, nonproductive binding of pesticides or their metabolites with the enzyme molecules, thereby decreasing the activity of LDH and/or blocking the enzyme synthesis^48, 49^.

### Carboxylesterase activity (CarE)

CarE hydrolyzes numerous endogenous and exogenous ester-containing compounds and participates in lipid transport and metabolism, which is important for the detoxification of many contaminants^50^. In *T. tubifex* (Fig. 7D), the CarE activities decreased significantly during early exposure periods (1, 5 and 9 days), thereafter increasing to levels comparable with the control group. The decrease might be an adaptive process, and then stimulated the detoxification metabolism. Significant decrease were observed on the 14th and 21st day in gill (Fig. 7E), while in hepatopancreas (Fig. 5F) and kidney (Fig. 7G) the CarE activities increased significantly. The elevation of CarE activities in hepatopancreas and kidney might be due to its role in the metabolism and detoxification of DDTs.

## Conclusions

This study is the first detailing the differences in accumulation, distribution, and elimination of DDTs in carp exposed in the context of food web, and the impact of DDTs on biochemical markers. *T. tubifex* could rapidly accumulate larger quantities of DDT, posing a risk to their predators, carp. The bioaccumulation curves of DDTs in *T. tubifex* and carp in the treatment group were different from those in the control group, due to the differences in DDT concentrations in the sediment. Tissue-specific accumulation was observed in carp, and the highest concentrations of DDTs was found in gill, followed by gonad and kidney. Analysis of the mass distribution of DDTs in the tissues/organs of carp indicated that gill, muscle and gastrointestinal tract were the main storage locations. Consumption of carp contaminated with DDTs at the levels observed in this study would not raise concerns for human health. The elimination of DDTs was observed in *T. tubifex*, but not in carp; however, tissue-specific elimination was observed in carp. Furthermore, our results provide evidence that DDTs induce physiological disturbances on biochemical markers in *T. tubifex* and carp tissues as well as causing oxidative stress and toxic effects. These findings have potential implications for risk assessment after contamination with DDTs in the context of aquatic food webs.

## Materials and Methods

### Ethics statement

Before initiating exposure experiments, approval was obtained from Animal Ethical committee at China Agricultural University. All methods were performed in accordance with the relevant guidelines and regulations.

### Standards and Reagents

Detailed information is provided in the Supplementary Material. DDT (o,p’-DDT, p,p’-DDT) and metabolites (o,p’-DDD, p,p’-DDD, o,p’-DDE, p,p’-DDE) were analyzed in the samples.

### Sediment preparation

The sediment was prepared in a glass aquarium with 3.6 kg soil (collected from China Agricultural University, and the physicochemical properties of the soil were as follows: clay, 34.8 ± 0.1%; silt, 10.0 ± 2.8%; sand, 55.2 ± 2.8%; organic matter, 24.4 ± 0.8 g/kg; and pH, 7.74 ± 0.05.) and 16 L de-chlorinated tap water, and incubated for seven days. The overlying water was discarded, and 400 g sediment (dry weight) was spiked with o,p’-DDT (50 ng/μL, 1600 μL) and p,p’-DDT (50 ng/μL, 1600 μL) in acetone. After the solvent was volatilized, the sediment was blended with the 3.6 kg sediment (dry weight) thoroughly. Dechlorinated tap water was added, and system was allowed to equilibrate for 48 h.

### Experimental design

Environmentally relevant concentration exposure was conducted according to Organization for Economic Cooperation and Development (OECD section 305 and 315) test guidelinesn^51, 52^. *Tubifex tubifex* (Oligochaeta, Tubificidae) was chosen as prey, which could accumulate DDTs from sediment and transfer them to carp (*Cyprinus carpio*). *T. tubifex* is one of the most widespread and ubiquitous groups, which was considered to be a good bio-indicator^53, 54^. Carp are omnivorous demersal fish in fresh water ecosystems, which can serve as bio-indicators of environmental contaminants and play significant roles in assessing potential risk^44^. Adult *T. tubifex* and carp (2.0 ± 0.2 g, about 5 cm in length, ignore the gender) were obtained from Guan Yuan market (Beijing, China) and acclimatized in the laboratory for 2 weeks. *T. tubifex* (80 g) and ten fish were added into the sediment-water system for the exposure experiment. Twenty-seven glass aquariums with spiked sediment were cultivated at 14 h/10 h photoperiods and at 20 ± 2 °C for 28 days (nine aquariums were used to analyze the individual fish and *T. tubifex* worms, eighteen aquariums were used to analyze the tissue distribution and toxic effects). The overlying water was gently aerated and unchanged. Overlying water, fish (one from each aquariums), worms (about 1 g) and sediment were sampled on the 1st, 3ed, 5th, 9th, 14th, 21st, and 28th day. At the end of bioaccumulation experiments, all the fish and worms were transferred to un-spiked sediment for elimination experiments, respectively. Overlying water, fish (one from each aquariums), worms (about 1 g) and sediment were sampled on the 1st, 3ed, 5th, 7th, and 10th day. The distribution and biological effects of DDTs in carp tissue were investigated in the bioaccumulation and elimination experiments. Fish were euthanized with an ice/water mixture. The blood, hepatopancreas, gastrointestinal tract, gallbladder, gonad, kidney, heart, gill, brain and muscle (triplicate, each contained three fish) were immediately sampled, and weighed for further toxicological analysis and DDTs measurements.

The control treatments (twenty-seven glass aquariums: nine aquariums were used to analyze the individual fish and *T. tubifex* worms, eighteen aquariums were used to analyze the tissue distribution and toxic effects) were composed of worms, fish and un-spiked sediment. The cultivation, sample time and dissection procedures were the same to the exposure experiments.

### Samples extraction and DDTs determination

The methodology of analyzing DDTs in worms, fish, overlying water and sediments were shown in the Supplementary Material. The different tissues/organs of carp were extracted with different methods. Details of the extraction process please refer to the Supplementary Material. The lipid content of animals was measured according to Bligh and Dyer^55^. DDTs concentrations in the worms, fish and sediment samples were analyzed with GC-μECD (Agilent Technologies Inc., USA). DDTs concentrations in the overlying water and tissues/organs samples were determined by GC-MS/MS (Thermo Fisher Scientific, USA). Details please refer to the Supplemental Material.

### Toxic effects assessment

In toxicological studies, changes in contents and enzyme activity often directly reflect cell damage in specific tissues/organs. The biochemical changes of the enzymatic defense systems can indicate the primary subcellular damage. Superoxide dismutase (SOD), catalase (CAT), glutathione-S-transferase (GST), total glutathione (GSH) content, lactate dehydrogenase (LDH) and carboxylesterase (CarE) were studied as biomarkers for toxic effects assessment.

The worms (1 g from three aquariums, triplicate) or tissues/organs (triplicate, each contained three fish) were homogenized in phosphate buffer (50 mM, pH 7.8) using Tissue Lyser at 4 °C (30 r/s, 3 min). The homogenate was centrifuged at 10 000 rpm for 10 min in a refrigerated centrifuge at 4 °C. The supernatant was diluted with 3 mL phosphate buffer and stored at −80 °C. The protein contents were measured using bovine serum albumin according to Bradford^56^ using UV-VIS spectrophotometer (UV-2600, SHIMADZU, Japan).

The CAT activity was measured by the decomposition rate of H_2_O_2_ (15 mM) at 240 nm in 3 mL volume at 20 °C for 1 min^57^. CAT values were expressed as U per mg of protein.

The LDH activity was measured by the reaction of tissue homogenate, 2.9 mL pyruvic acid solution (0.98 mM) and 100 μL NADH (5.3 mM) at 340 nm for 3 min. The NADH and pyruvic acid solution was made with phosphate buffer (0.1 M, pH 7.5) at 25 °C. The change rate of absorbance (ΔA_340nm/min_) was calculated when the absorbance changed linearly. The LDH activity was calculated by LDH (U/mg protein) = ΔA_340nm/min_ × dilution ratio/tissue homogenate volume (mL)/protein content (mg protein/mL).

SOD, GST, GSH and CarE were measured using commercial test kits (Nanjing Jiancheng Bioengineering Institute, Suzhou Comin Biotechnology Co., Ltd.). The unit of SOD was the amount of enzyme needed to inhibit 50% of the reaction solution (1 mL) per mg of protein (U/mg protein). The unit of GST was the amount of enzyme needed to reduce 1 μmol/L GSH at 37 °C in 1 min per mg of protein (U/mg protein). The contents of GSH were determined by the reaction of DTNB and sulfhydryl compound (mg GSH/g protein). The unit of CarE was the amount of enzyme needed to catalyze increase in absorbance of 1 at 37 °C in 1 min per mg of protein (U/mg protein).

### Data analysis

The analysis of difference in our experiment was conduct by SPSS 20.0 (One-way ANOVA, S-N-K test).

## Electronic supplementary material

Supplementary Material
