# Supplementary material for: Assessment of tissue-specific accumulation, elimination and toxic effects of dichlorodiphenyltrichloroethanes (DDTs) in carp through aquatic food web
Source: Sci Rep. 2017 May 23;7:2288. doi: 10.1038/s41598-017-02612-4 (PMC5442124; doi:10.1038/s41598-017-02612-4)
Supplement: Supplementary file 1 — Supplementary Material [file 41598_2017_2612_MOESM1_ESM.pdf]

Supplementary Material

Assessment of tissue-specific accumulation, elimination and toxic effects of dichlorodiphenyltrichloroethanes (DDTs) in carp through aquatic food web

Shanshan Di[1,2], Ruiquan Liu[2], Zhongnan Tian[2], Cheng Cheng[2], Li Chen[1,2], Wenjun Zhang[1,2], Zhiqiang Zhou[1,2], Jinling Diao[2]*

[1] Beijing Advanced Innovation Center for Food Nutrition and Human Health, Department of Applied Chemistry, China Agricultural University, Yuanmingyuan west road 2, Beijing 100193, China

[2] Department of Applied Chemistry, China Agricultural University, Yuanmingyuan West Road 2, Beijing, 100193, China

*Corresponding author:

Jinling Diao, Department of Applied Chemistry, China Agricultural University, Yuanmingyuan west road 2, Beijing 100193, P.R. China;

Tel: +86 13811992249; Fax: +8610-62733547;

E-mail:lingyinzi1201@gmail.com

Number of Pages: 9

Number of Tables: 1

Number of Figures: 6

Standards and Reagents. DDTs (p,p'-DDT, o,p'-DDT, p,p'-DDD, o,p'-DDD, p,p'-DDE, o,p'-DDE) and PCB-209 (recovery surrogate) were purchased from J&K Scientific Ltd. Acetone, dichloromethane, chloroform, methanol, *n*-hexane, petroleum ether and anhydrous sodium sulfate (Na_2SO_4) were analytical grade. Sulfuric acid (H_2SO_4, 98%) and silica gel (200–300 mesh) were purchased from Sinopharm Chemical Reagent Co., Ltd. Chromatographic-grade isooctane was purchased from Merck. Sulfuric acid-impregnated silica was prepared in the laboratory mixing neutral silica gel (10 g, previously baked at 150 ℃ for 3 hours) with sulfuric acid (4 mL, 98%).

Samples extraction and DDTs determination. The water samples (20 g) were extracted twice with dichloromethane (20 mL) by oscillating. The extraction solvent was collected and concentrated to 5-10 mL at 30 ℃. The concentrate was concentrated with a stream of nitrogen in a test tube, and re-dissolved with 200 μL isooctane.

Worms and individual fish samples (0.5 g, PCB-209 was added as recovery surrogate) were homogenized with Tissue Lyser (MM400, Retsch GmbH, Germany) for 3 min at 30 r/s. Acetone and petroleum ether (1:1, V/V) was added and extracted 3 times with Tissue Lyser (30 r/s, 3 min). The mixture was centrifuged at 10 000 rpm for 3min. The extracting solution was merged and transferred to a 5 mL centrifuge tube. The Florisil-SPE cartridge (1000 mg, 6 mL, Agilent SampliQ Products) and 3 g sulfuric acid-impregnated silica was used to clean up interfering substances. The cartridge was eluted with 10 mL of dichloromethane, 5 mL of n-hexane, and then equilibrated with 5 mL leachate (dichloromethane: n-hexane=2: 8, v/v). The extracting solution was loaded to the cartridge, eluting with 10 mL leachate. The eluent was collected in a glass tube, concentrated to dryness under a gentle nitrogen flow, and diluted with 200 μL of isooctane.

Microwave-assisted solvent extraction (MAE) was used to extract the sediment samples (5 g) with acetone and petroleum ether (1:1, V/V, 20 mL) at 100 ℃ for 10 min. After dehydrating and concentrating to dryness, the purification method was the same as above. 200 μL of isooctane was used to reconstitute the sediment extract.

The different tissues/organs of carp were extracted with different methods. The methodology of analyzing DDTs in kidney and muscle samples were same with worms. Hepatopancreas and gastrointestinal tract samples were extracted with acetone and petroleum ether (1:2, V/V), the purification method was the same to kidney and muscle samples. The extraction method of blood, heart, gallbladder and gonad samples was the same as kidney and muscle samples, while purification was not needed. Matrix solid-phase dispersion was used to extract gill and brain samples. Adding sample, PCB-209, 1 g anhydrous sodium sulfate (Na_2SO_4) and 2 g sulfuric acid-impregnated silica to a glass mortar, the mixture was thoroughly ground with a glass pestle until it became dry and homogeneous, which was transferred into a Florisil-SPE cartridge. The cartridge was eluted with 12 mL eluent (dichloromethane: n-hexane=2: 8, v/v). The eluent was concentrated in a gentle nitrogen flow, and reconstituted with 200 μL of isooctane.

The lipid content of organism tissues were measured according to Bligh and Dyer[1]. Chloroform, methanol and water (1:2:0.8) was used to extract the lipid, and then changed the extracting solution

proportions to 2:2:1.8 to separate lipid. Lipid was in the chloroform layer. The lipid contents in worm and fish tissues at the end of the experiment were not differ from those at the start by more ± 10%.

Agilent 7890A gas chromatograph (GC-μECD) with a HP-5 column (30 m, 0.25 mm i.d., 0.25 μm film thickness; Agilent Technologies Inc.) was used to analyze worms, fish and sediment samples. High-purity N_2 was used as carrier gas (1 mL/min). The injector and detector temperatures were 270 ℃ and 290 ℃, respectively. The oven temperature program was as follows: initial temperature began at 70 ℃ (held for 1 min), increased at 20 ℃/min to 200 ℃ (held for 5 min), and finally increased at 5 ℃/min to 285 ℃ (held for 10 min). One microliter of sample solution was injected in splitless mode.

The identities of DDTs in the other samples were confirmed using a GC-MS/MS (Thermo Fisher Scientific, USA) with a HP-5MS column (30 m, 0.25 mm i.d., 0.25 μm film thickness; Agilent Technologies Inc.). Helium was used as carrier gas (1.0 mL/min). The injector and transmission line temperatures were 270 ℃ and 250 ℃, respectively. GC conditions were as follows: initial column temperature 90 ℃ (2 min), increased at 10 ℃ /min to 200 ℃ (held for 5 min), and then 10 ℃ /min to 290 ℃ (held for 6 min). The MS/MS conditions and selected ions (m/z) were shown in Supplemental Material Table S1.

The average recoveries for DDTs in different organ/tissues ranged from 64.3 % to 128.2 % with SD below 20 %. The average recoveries for DDTs in worms, fish, overlying water and sediment ranged from 62.9 % to 113.1 % with SD below 20 %.

1 Bligh, E. G. & Dyer, W. J. A rapid method of total lipid extraction and purification. *Canadian Journal of Biochemistry and Physiology* **37**, 911-917 (1959).

Table S1. The MS/MS conditions and selected ions (*m/z*) of PCB-209 and DDTs.

Pesticides	Parent/Product ion(m/z)		SRM Collision Energy		Retention Time
o,p'-DDE	246/176	318/246	25	20	19.51
p,p'-DDE	246/176	318/246	25	20	20.61
o,p'-DDD	235/165	237/165	20	20	20.87
p,p'-DDD	235/165	237/165	20	20	21.86
o,p'-DDT	235/165	237/165	20	20	21.95
p,p'-DDT	235/165	237/165	20	20	22.87
PCB-209	496/426	498/428	25	25	27.73

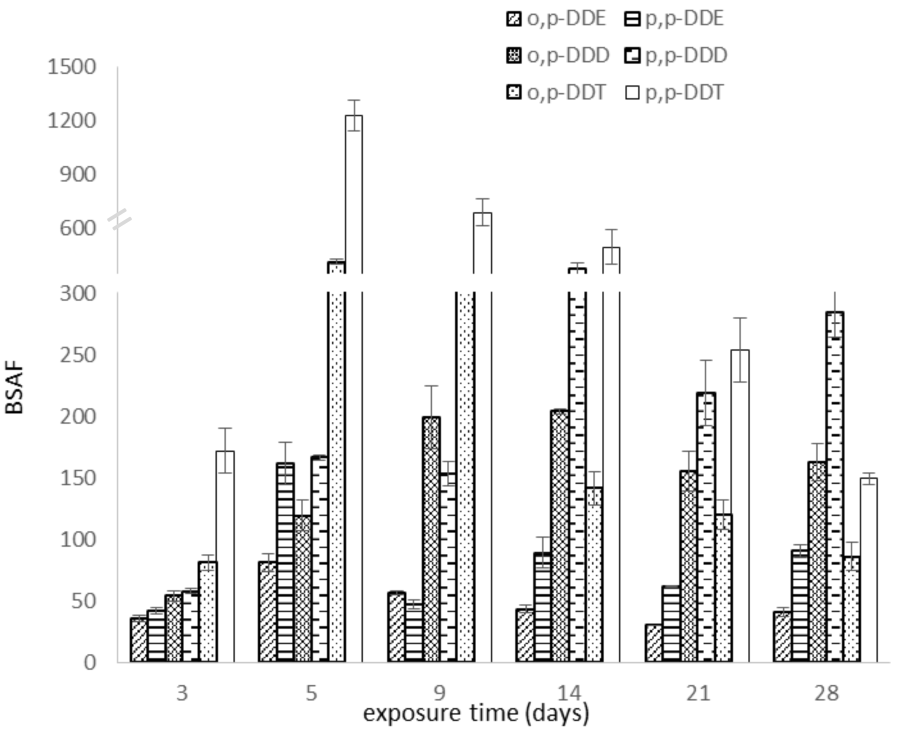

Figure S1. The BMF values of DDTs in food web in the treatment group.

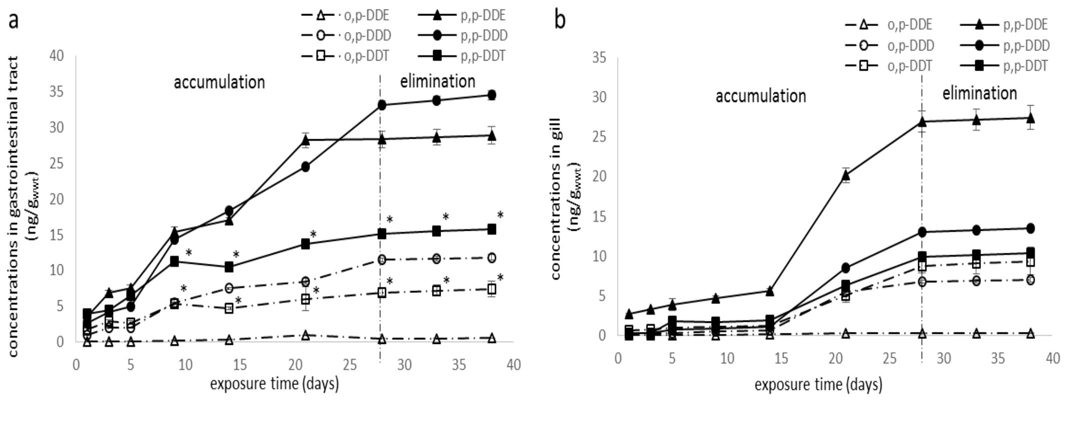

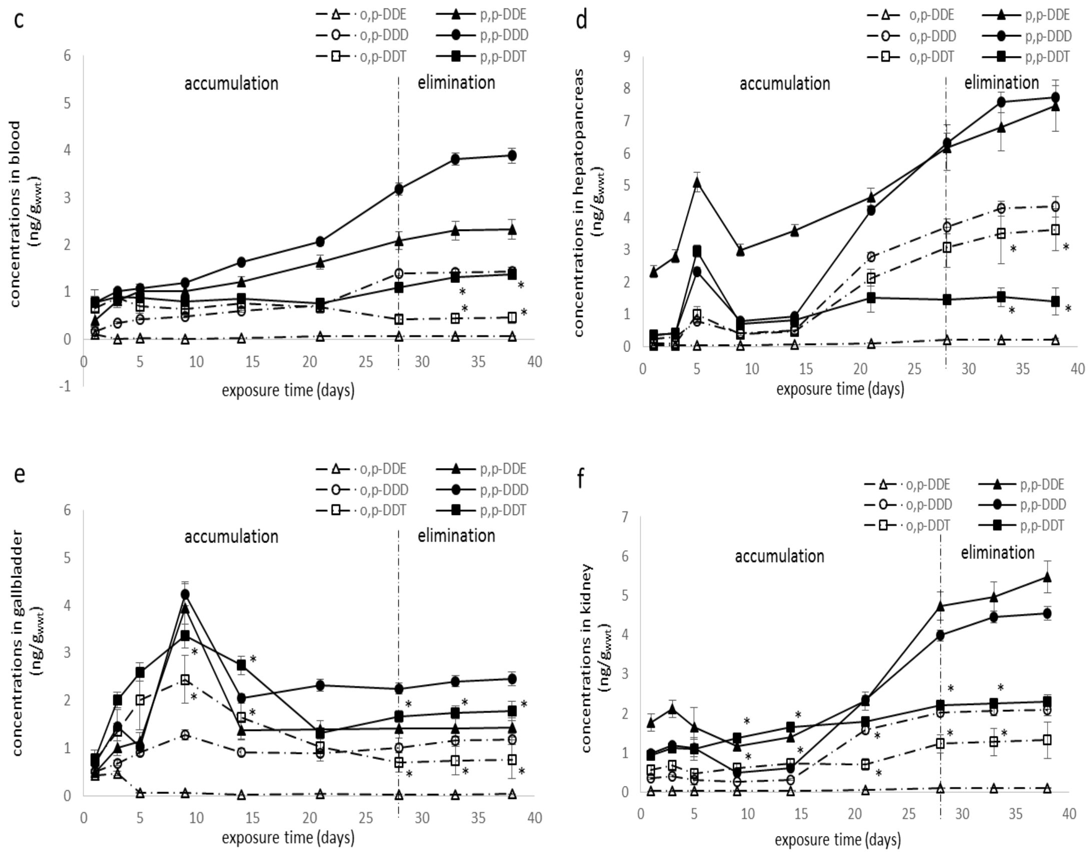

Figure S2. The concentrations of DDTs in gastrointestinal tract (a), gill (b), blood (c), hepatopancreas (d), gallbladder (e) and kidney (f) in carp in the control group in the accumulation and elimination experiments. * indicates significant difference between p,p'-DDT and o,p'-DDT ($p < 0.05$, S-N-K test).

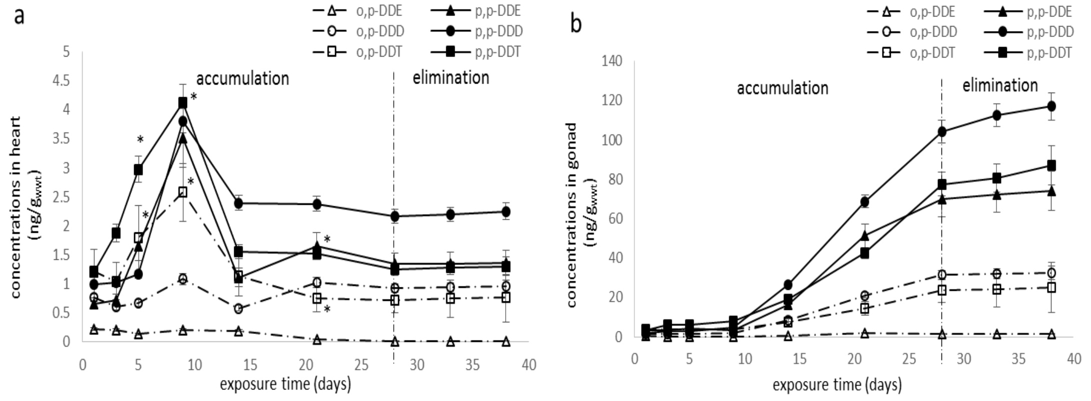

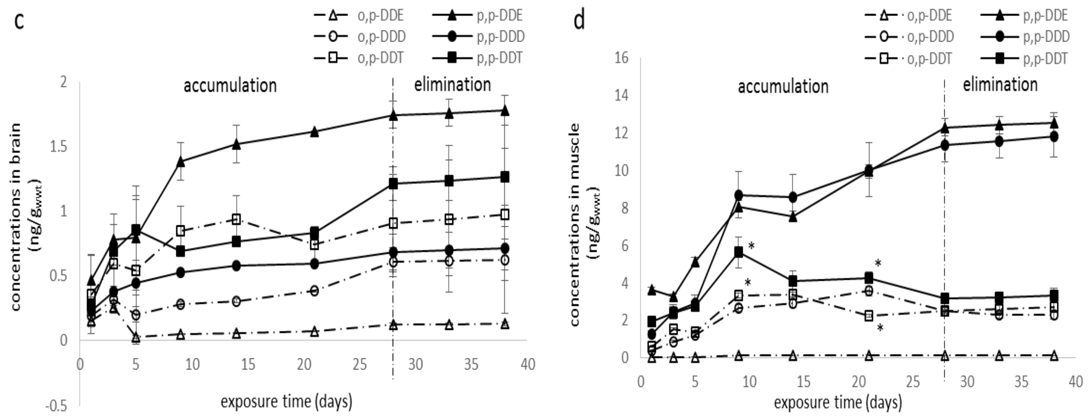

Figure S3. The concentrations of DDTs in heart (a), gonad (b), brain (c) and muscle (d) in carp in the control group in the accumulation and elimination experiments. * indicates significant difference between p,p'-DDT and o,p'-DDT ($p < 0.05$, S-N-K test).

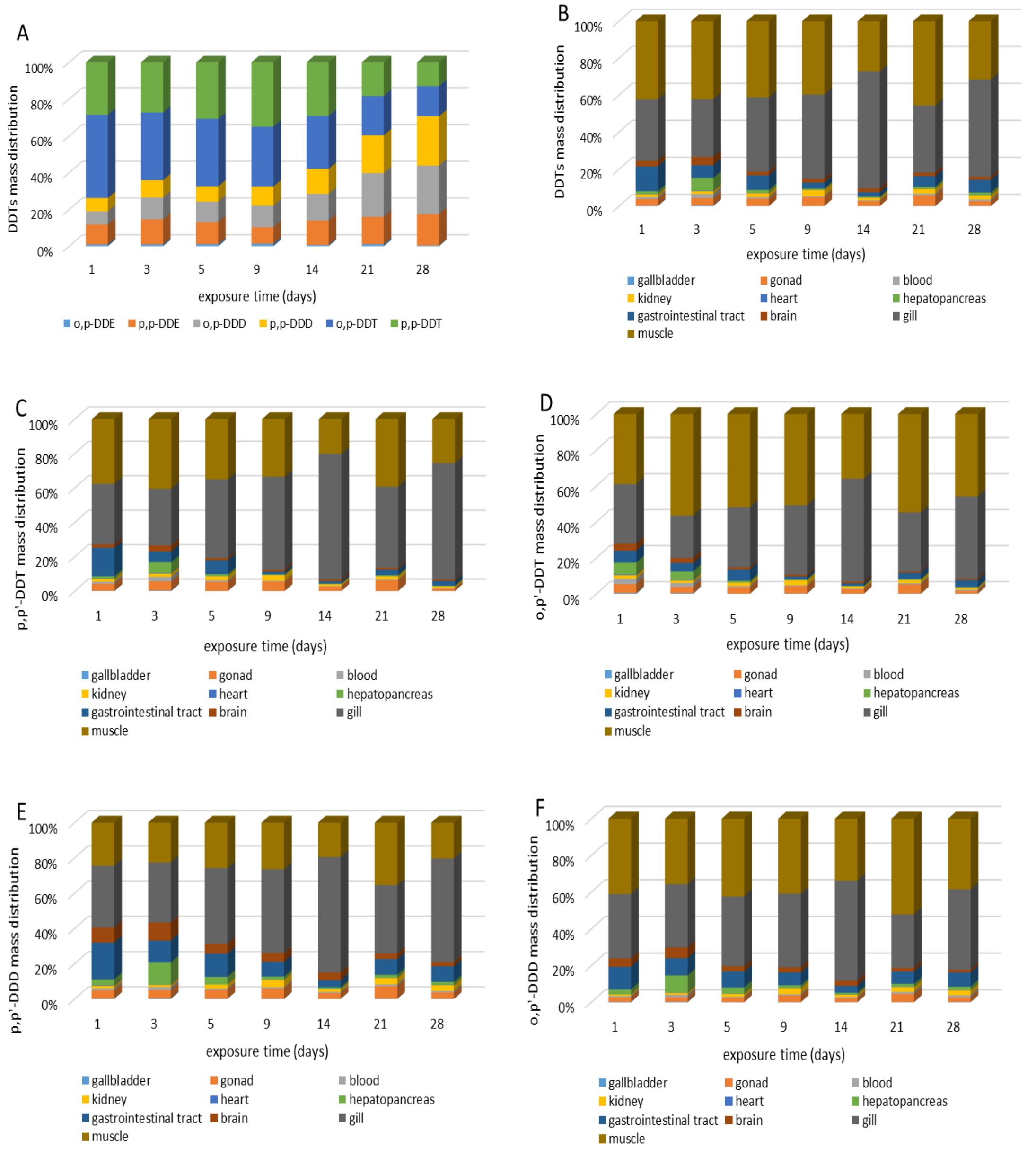

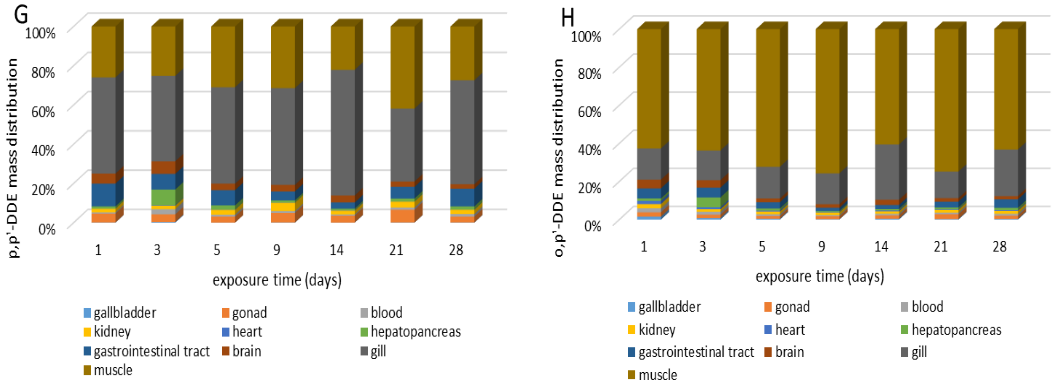

Figure S4. The mass distribution of DDTs in carp in the treatment group (A). The mass distribution of total DDTs in the tissue/organ of carp in the treatment group (B). The mass distribution of p,p'-DDT (C), o,p'-DDT (D), p,p'-DDD (E), o,p'-DDD (F), p,p'-DDE (G), o,p'-DDE (H) in the tissue/organ of carp in the treatment group.

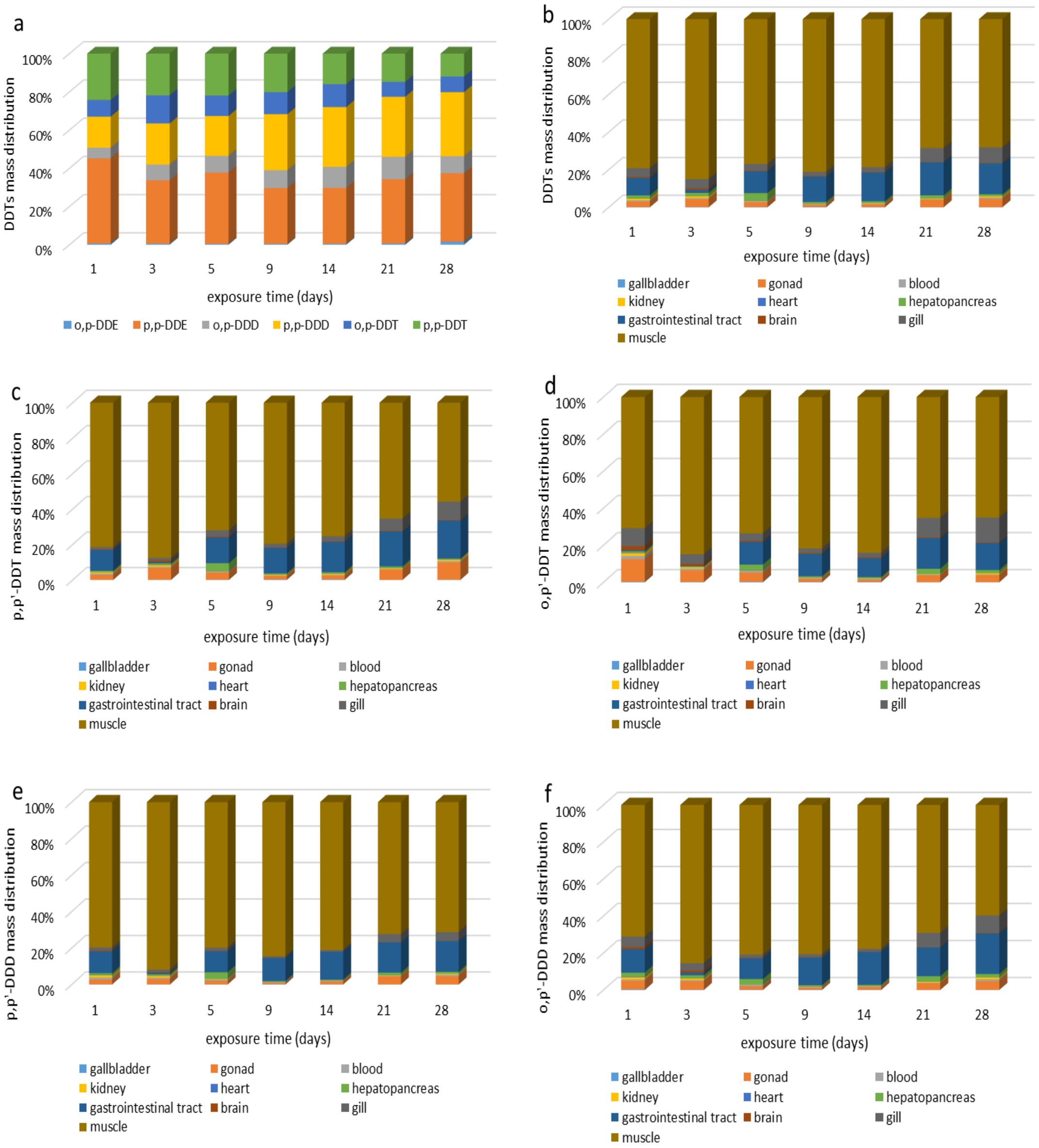

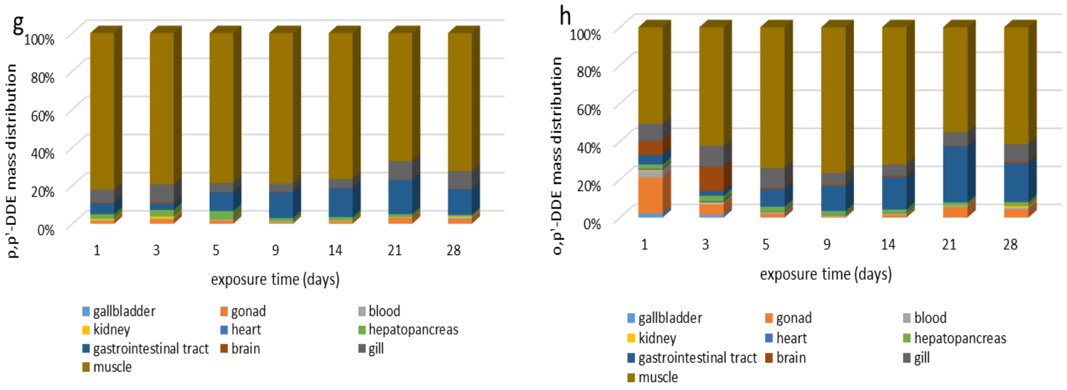

Figure S5. The mass distribution of DDTs in carp in the control group (a). The mass distribution of total DDTs in the tissue/organ of carp in the control group (b). The mass distribution of p,p'-DDT (c), o,p'-DDT (d), p,p'-DDD (e), o,p'-DDD (f), p,p'-DDE (g), o,p'-DDE (h) in the tissue/organ of carp in the treatment group and control group.

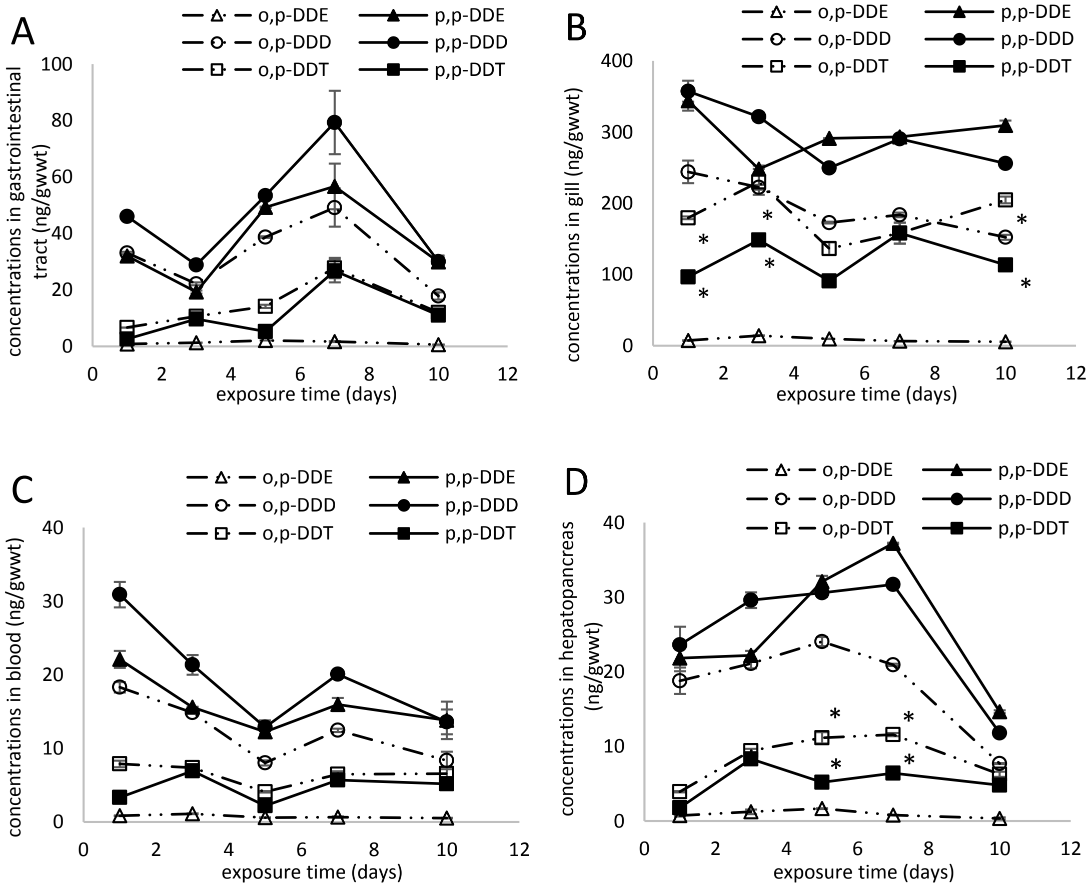

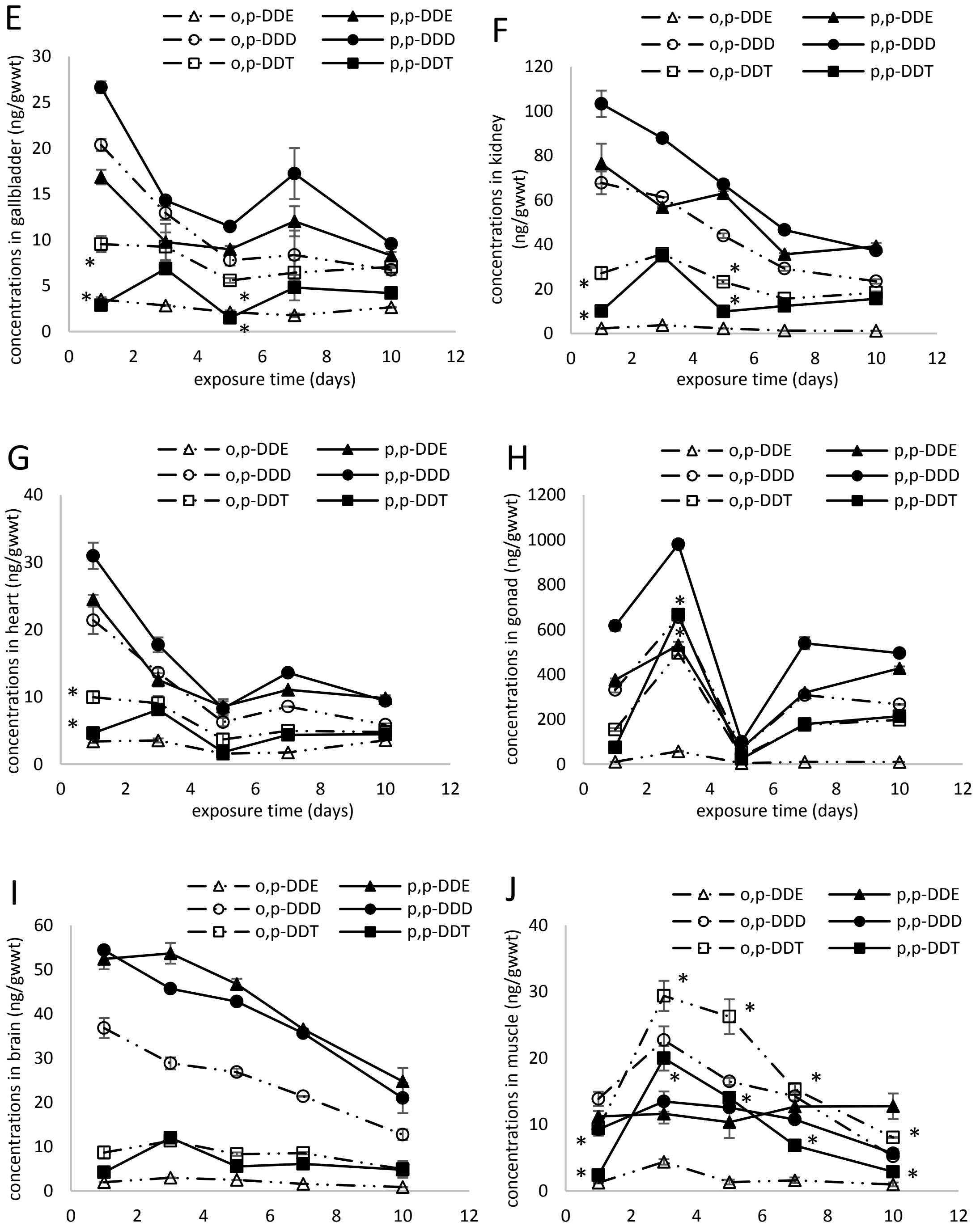

Figure S6. The elimination of DDTs in gastrointestinal tract (A), gill (B), blood (C), hepatopancreas (D), gallbladder (E), kidney (F), heart (G), gonad (H), brain (I) and muscle (J) in carp in the treatment group. * indicates significant difference between p,p'-DDT and o,p'-DDT (p < 0.05, S-N-K test).
